# Osteoimmune senescence in aging bone: from inflammaging dogma to cell-type-specific therapeutic windows

**DOI:** 10.3389/fimmu.2026.1940235

**Published:** 2026-09-22

**Authors:** Lan Zhang, Yao Yao, Jinxu Chen

**Affiliations:** 1 Department of Pharmacy, Tongde Hospital of Zhejiang Province Affiliated to Zhejiang Chinese Medical University, Hangzhou, China; 2 Department of Pharmacy, Women’s Hospital, Zhejiang University School of Medicine, Hangzhou, China; 3 Department of Traditional Chinese Internal Medicine, Tongde Hospital of Zhejiang Province Affiliated to Zhejiang Chinese Medical University, Hangzhou, China

**Keywords:** aging-related bone diseases, bone marrow niche, cellular senescence, inflammaging, osteoimmune senescence

## Abstract

Aging-related bone diseases are often interpreted through hormonal decline, defective remodeling, and chronic low-grade inflammation, yet these accounts do not fully capture the cellular, spatial, and temporal heterogeneity of skeletal aging. Bone is an immune-active organ sustained by coordinated signaling among skeletal, hematopoietic, immune, adipose, vascular, and neural compartments. Cellular senescence perturbs this network through stable cell-cycle arrest, metabolic remodeling, and a heterogeneous senescence-associated secretory phenotype (SASP). Whereas recent osteoimmunology syntheses broadly catalog age-related immune–bone interactions, our specific advance is to treat p16- and p21-associated states as functionally non-equivalent and to translate cell identity, anatomical compartment, persistence, and disease stage into explicit therapeutic decision points. We compare senescence programs in bone marrow stromal cells, osteoblast-lineage cells, osteocytes, osteoclast precursors, and immune populations, and then place them within an aging marrow ecosystem shaped by hematopoietic drift, marrow adiposity, vascular decline, and neural degeneration. These mechanisms are mapped across osteoporosis, osteoarthritis, rheumatoid arthritis, periodontitis, and delayed fracture healing. Emerging interventions include senolytics, senomorphics, immune-mediated clearance, bone-targeted delivery, and biomarker-guided patient selection. By operationalizing cell- and stage-resolved therapeutic windows, this framework aims to move senescence-targeted treatment beyond broad senolysis and toward testable precision strategies for aging bone.

## Graphical Abstract

**Figure:**
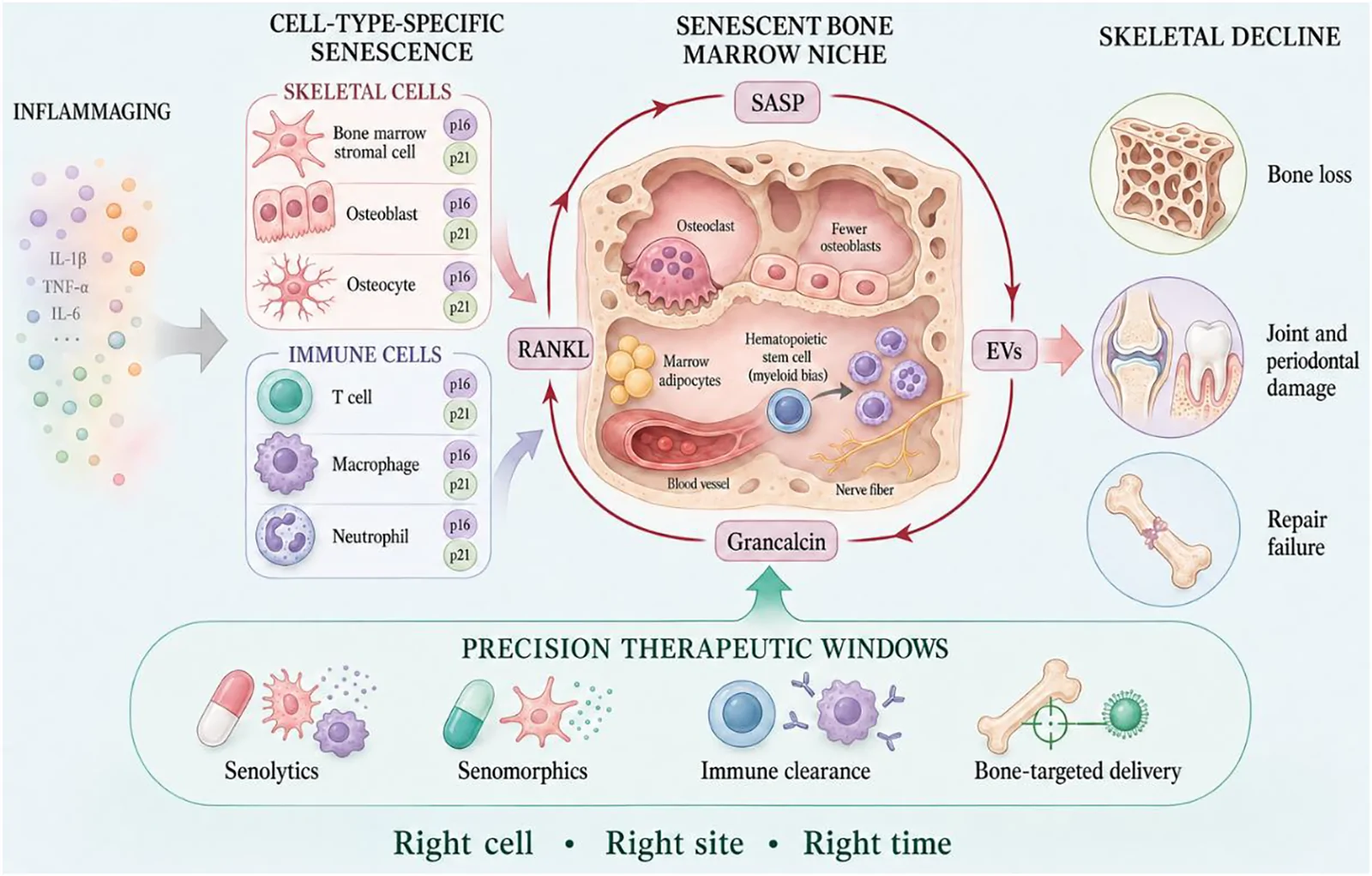

## 1 Introduction

Population aging has made skeletal degeneration an increasingly important cause of disability, loss of mobility, and dependence. Osteoporosis, osteoarthritis, periodontitis, and delayed fracture healing have traditionally been attributed to hormonal decline, cumulative mechanical stress, or defective bone remodeling. These factors remain relevant, but they are no longer sufficient to explain the biology of the aging skeleton. Bone is an immune-active organ in which osteoblasts, osteoclasts, osteocytes, stromal cells, hematopoietic cells, and immune populations continuously exchange regulatory signals. Aging disturbs these interactions and establishes a persistent, low-grade inflammatory milieu classically described as inflammaging (1).

Recent epidemiological data underscore the scale of the problem. A 2021 meta-analysis estimated the global prevalence of osteoporosis at 18.3% (95% CI, 16.2–20.7) (2). In the nationally representative China Osteoporosis Prevalence Study, 5.0% of men and 20.6% of women aged 40 years or older had osteoporosis by dual-energy X-ray absorptiometry, while vertebral fracture prevalence was 10.5% in men and 9.7% in women (3). The burden is also economic: an analysis of 39,300 fracture hospitalizations in Jiangsu Province reported mean inpatient costs ranging from US$3,142 for hand or wrist fractures to US$10,355 for hip fractures in 2016 US dollars (4). Because these estimates exclude much outpatient care, rehabilitation, informal caregiving, and productivity loss, they probably underestimate the full societal cost of skeletal aging.

The inflammaging paradigm has been valuable because it connects elevated inflammatory mediators in aged bone with enhanced osteoclastogenesis, impaired osteogenesis, and declining regenerative capacity (5). Recent work supports a bidirectional relationship. Chronic inflammatory and oxidative stress can induce senescence (6, 7). In turn, SASP-producing skeletal, stromal, adipose, and immune cells can maintain local and systemic inflammation (8, 9). Senescent cells accumulate within the aging bone microenvironment, and their genetic or pharmacological clearance prevents age-related bone loss in mice (10). Senescence is therefore more than a biomarker of chronological aging; it can actively reshape skeletal homeostasis. Through SASP signaling and impaired immune surveillance, cellular senescence links immune aging to unbalanced remodeling, defective repair, and a spectrum of age-related bone diseases (**Figure 1**).

**Figure 1:**
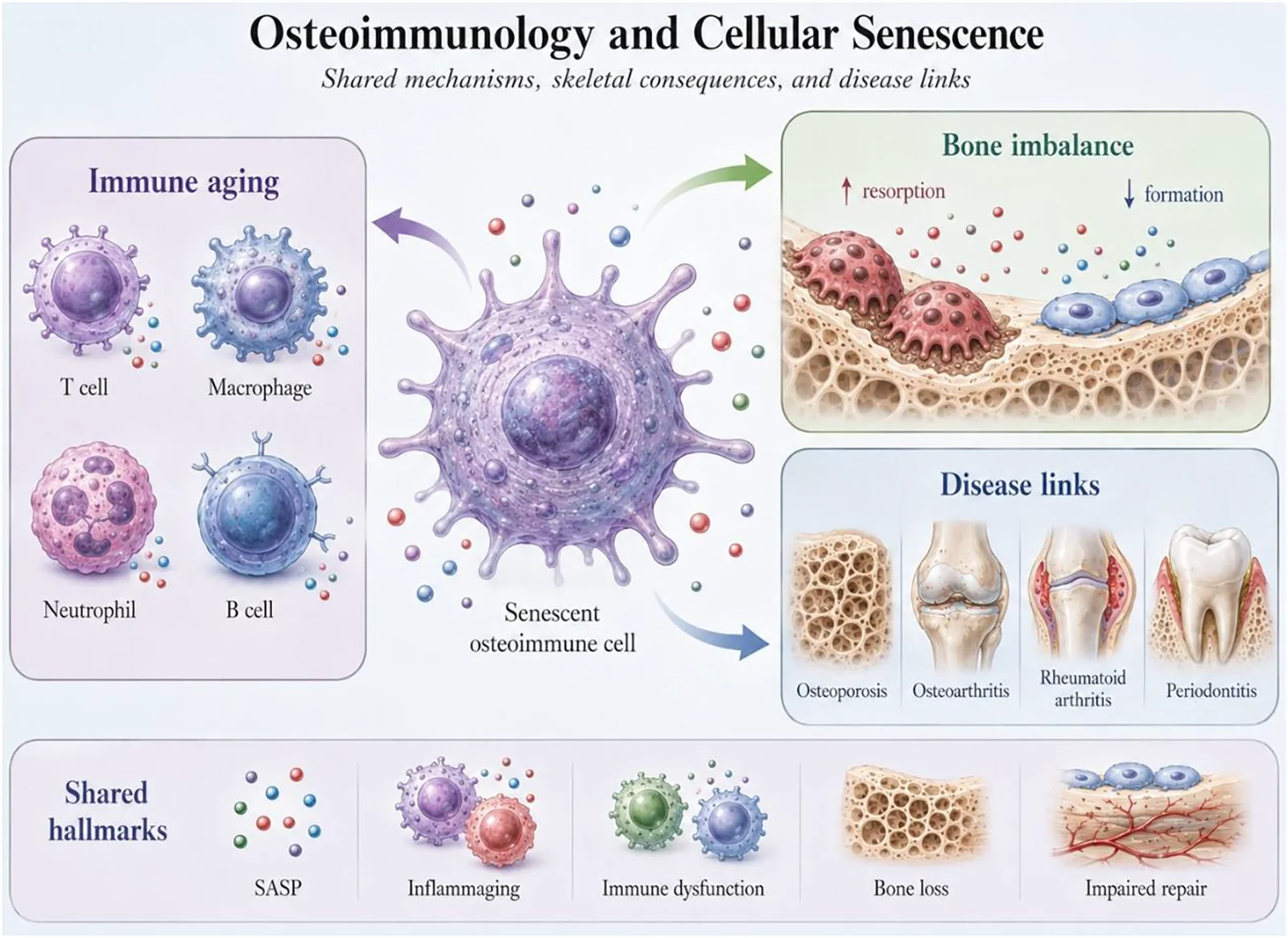
Osteoimmunology and cellular senescence: shared mechanisms, skeletal consequences, and disease links. Aging and cellular senescence alter the functions of major immune populations, including T cells, B cells, macrophages, and neutrophils. Senescent osteoimmune cells release SASP factors and other inflammatory mediators that sustain inflammaging and immune dysfunction. These signals disrupt the coupling of bone remodeling by enhancing osteoclast-mediated resorption while suppressing osteoblast-mediated formation. The resulting osteoimmune imbalance promotes bone loss and impairs skeletal repair. Although the dominant cells and tissue compartments differ among diseases, these interconnected processes contribute to osteoporosis, osteoarthritis, rheumatoid arthritis, and periodontitis. All elements in this figure were originally drawn by the authors using Adobe Illustrator.

Nevertheless, reducing skeletal aging to generalized inflammation conceals substantial biological heterogeneity. The consequences of senescence vary with cell lineage, initiating stress, anatomical compartment, disease phase, and the efficiency of immune clearance. Even the SASP is not a fixed inflammatory output; it evolves over time and differs across cellular contexts (6). In bone, these distinctions are consequential. Senescent osteocytes can promote RANKL-dependent cortical bone loss (11), whereas senescent stromal cells lose osteogenic competence and favor marrow adiposity (7). Senescence-associated immune states may also disturb skeletal homeostasis through mediators such as grancalcin, but cellular attribution is context-specific: Li et al. implicated aged marrow neutrophils and macrophages in chronic skeletal aging, whereas Zou et al. identified aged macrophages as the relevant source during fracture repair (12, 13).

Against this background, we use osteoimmune senescence as a more discriminating framework for aging-related bone disease. Rather than treating inflammaging as a uniform process, the review resolves senescence across specific skeletal and immune lineages and places these cells within an integrated marrow ecosystem that includes hematopoiesis, adiposity, vasculature, and neural regulation. A 2025 review recently synthesized the broad mechanisms and therapeutic implications of osteoimmunology in aging (14). Our increment is narrower and operational: we center the demonstrated non-equivalence of p16- and p21-associated populations and use cell identity, compartment, persistence, and disease stage to define testable intervention windows. For example, p21-positive and p16-positive populations exert different effects after radiation injury and during fracture repair (15, 16), while intermittent dasatinib plus quercetin failed to reduce bone resorption across an unselected cohort of postmenopausal women (17). The translational problem is therefore not simply how to eliminate senescent cells, but how to identify the pathogenic population, tissue compartment, and disease stage in which intervention is warranted. This explicit distinction between marker-defined programs and stage-specific treatment logic is the organizing advance of the review.

### 1.1 Aging remodels the marrow, immune, and skeletal baseline

Before individual senescence programs are considered, the age-associated baseline must be defined. Across recent human and murine studies, aging reduces stromal clonogenic and osteogenic capacity, shifts marrow stromal fate toward adipocytes, remodels inflammatory stromal states, reduces lymphoid progenitor output, and favors differentiated or myeloid-biased hematopoietic states. Bone formation declines while marrow adiposity and pro-resorptive signaling rise. **Table 1** summarizes representative studies from the past 10 years. Because species, age bins, tissues, and assays differ, the direction and context of change are more comparable than raw effect sizes.

**Table 1:** Representative studies of age-associated changes in marrow, immune, and skeletal cell populations published within the past 10 years.

Cell population	Study type	Sample size/groups	Methods/markers	Age-related changes	Ref.
Human BM-MSCs	Ex vivo; cross-sectional	Young donors n=28, ages 1–17 y (mean 9); older donors n=20, ages 55–92 y (mean 64); assay-specific n varied	ISCT flow phenotype, CFU-F, population doubling, SA-β-gal, ROS, RT-qPCR (p16, p21, p53), cytokine ELISA	Older BM-MSCs were larger and more granular, with increased SA-β-gal, ROS, p16, p21, IL-6/IL-8 and slower growth; clonogenicity declined; p53 transcript did not differ	(18)
Marrow immune populations (T, B, NK, monocytes/macrophages)	*In vivo*; cross-sectional mouse study	SPF mice: 6 mo, n=6; 20 mo, n=13	Bone marrow and spleen CyTOF, manual gating, t-SNE, cytokine profiling	Aged marrow showed more effector/resident-memory T cells and monocytes, fewer pro-/pre-B cells and naïve CD62L+ NK cells, and more differentiated PD-L1+ macrophage states	(19)
LepR+ periarteriolar stromal cells and lymphoid progenitors	*In vivo*; mouse multi-omic and perturbation study	Ages 2, 12, and 24 mo; n=3/age for stromal scRNA-seq; independent validation cohorts	scRNA-seq, flow cytometry, immunofluorescence, spatial localization, interferon-pathway perturbation	Sca1+Cxcl9+LepR+ inflammatory periarteriolar stromal cells expanded with age, while adjacent lymphoid progenitors were depleted; interferon blockade partly rescued the phenotype	(20)
HSCs	*In vivo*; transplantation and molecular profiling	Female mice: young donors 10 wk vs aged donors 20 mo; assay-specific cohort sizes reported for each experiment	Competitive transplantation, flow phenotyping, transcriptome and DNA-methylation profiling	Aged HSCs retained impaired regenerative output and myeloid bias in a young niche despite partial transcriptional rejuvenation, supporting both intrinsic and niche contributions	(21)
TGFβ1+CCR5+ marrow neutrophils	*In vivo*; mouse phenotyping, transfer, and pharmacological perturbation	Male mice across 2–24 mo; assay-specific cohort sizes reported for each experiment	Flow cytometry, micro-CT, adoptive transfer, TRAF3/TGFβ1 pathway assays, CCR5 inhibition	The subset expanded with age and was linked experimentally to bone loss; maraviroc reduced its abundance and increased bone mass in aged mice	(22)
Bone marrow adipocytes	*In vivo* and ex vivo; mouse aging and genetic perturbation	Young vs aged mouse cohorts; assay-specific cohort sizes reported for each endpoint	Lineage analysis, histology, micro-CT, senescence markers, PCLAF perturbation	Marrow adipocyte senescence and PCLAF expression increased with age; reducing this program attenuated skeletal aging phenotypes	(23)
Osteocytes	*In vivo* and *in vitro*; mouse genetics and mechanobiology	Young vs aged mice plus osteocyte-specific Tln1 and p53 knockout cohorts; assay-specific cohort sizes reported for each endpoint	Micro-CT, histomorphometry, RNA-seq, immunostaining, co-immunoprecipitation, mechanical loading	Talin1 declined with age; talin1 loss increased nuclear p53 and p16/p21-associated senescence, while osteocyte p53 deletion rescued bone loss and mechanoadaptation	(24)

BM-MSC, bone marrow mesenchymal stromal cell; CFU-F, colony-forming unit–fibroblast; CyTOF, cytometry by time of flight; HSC, hematopoietic stem cell; ISCT, International Society for Cell & Gene Therapy; ROS, reactive oxygen species; SA-β-gal, senescence-associated β-galactosidase; scRNA-seq, single-cell RNA sequencing; SPF, specific pathogen-free. Exact sample sizes are shown for single-cohort studies; multi-arm studies used assay-specific cohort sizes reported with the corresponding experiments in the cited articles.

## 2 Beyond the inflammaging dogma: why senescence in bone is context-dependent

The central limitation of the inflammaging model is that it describes a tissue state without resolving the cells that create, sustain, or respond to it. In aging bone, senescence may arise in osteocytes, osteoblast-lineage cells, marrow stromal cells, osteoclast precursors, and immune populations, with consequences that depend on the inducing stress and local niche. The SASP can be destructive, adaptive, or reparative, and its biological meaning changes with duration and clearance (6). Recent bone- and osteoimmune-focused syntheses likewise emphasize that cellular source, niche, and temporal state condition these effects (7, 8, 14). Osteoimmune senescence is therefore better understood as a set of cellular programs superimposed on chronic inflammation, rather than as a synonym for inflammaging. Cell identity and persistence jointly shape skeletal outcomes and opportunities for senolytic, senomorphic, or immune-mediated intervention (**Figure 2**).

**Figure 2:**
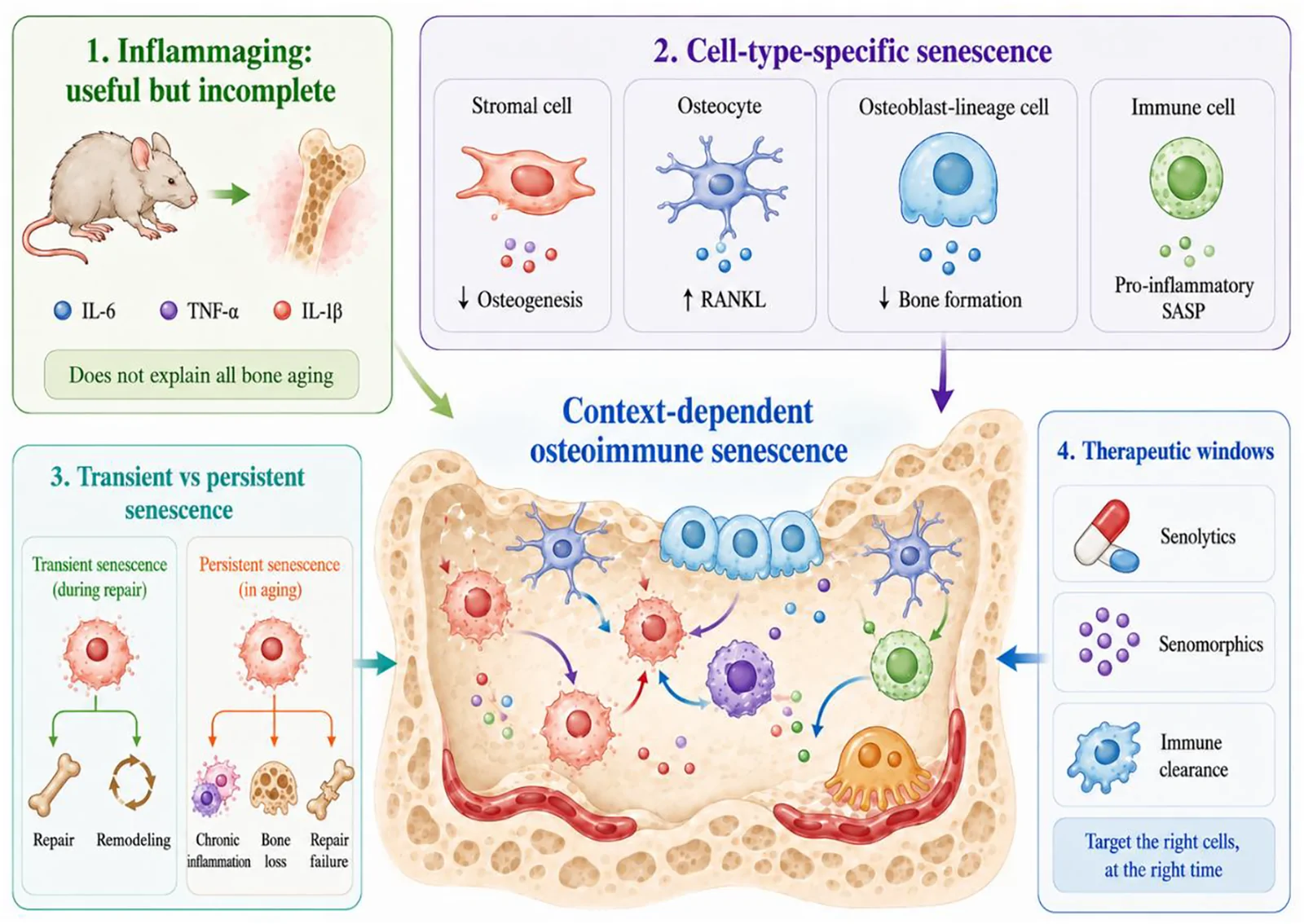
Context-dependent osteoimmune senescence in aging bone. Inflammaging provides an important but incomplete explanation for skeletal aging. Aging bone is shaped by multiple senescent cell populations, including stromal cells, osteocytes, osteoblast-lineage cells, and immune cells. These cells produce distinct senescence-associated signals and exert different effects on osteogenesis, bone formation, RANKL-mediated osteoclastogenesis, and inflammatory remodeling. The biological outcome of senescence also depends on timing. Transient senescence may participate in repair and remodeling, whereas persistent senescence promotes chronic inflammation, bone loss, and repair failure. This context-dependent framework supports a shift from broad anti-inflammatory or senolytic strategies toward precision therapeutic windows, including senolytics, senomorphics, and immune-mediated clearance, with the goal of targeting the right senescent cells at the right time. All elements in this figure were originally drawn by the authors using Adobe Illustrator.

### 2.1 Inflammaging is a useful but incomplete model

Inflammaging was originally proposed to describe the persistent inflammatory tone that accompanies aging and immunosenescence (1), and it has strongly influenced current models of age-related skeletal disease. The concept is consistent with the inflammatory signaling and reduced regenerative capacity observed in aged bone (5), as well as with the accumulation of senescent cells in the skeletal microenvironment (10). Genetic and pharmacological clearance experiments subsequently established that these cells can actively drive bone loss, providing a rationale for senescence-directed treatment in osteoporosis (25). Yet inflammaging is a background condition rather than a complete mechanism. It does not distinguish inflammatory signals by cellular source or function, and it can encourage the assumption that all senescent cells are deleterious and should be removed. In fact, DNA damage, mitochondrial dysfunction, oncogenic signaling, radiation, mechanical stress, and chronic inflammation can each induce senescence (26), potentially generating phenotypes with different secretomes, survival dependencies, and effects on surrounding tissue. Mechanistic interpretation requires cellular resolution beyond the inflammatory milieu itself.

### 2.2 The SASP is heterogeneous and cell-type dependent

The SASP is a principal route through which senescent cells modify their surroundings, comprising cytokines, chemokines, growth factors, proteases, and matrix-remodeling enzymes (27). Its composition varies with cell type, senescence inducer, tissue environment, and time since arrest (6). This diversity is particularly relevant in bone: osteocyte-derived signals can alter RANKL expression and local osteoclast activity (11), stromal-cell senescence can suppress osteogenesis while promoting marrow adiposity (5, 8), and immune-cell aging can remodel osteoclastogenic cytokine networks. Although these outputs may overlap, their origin determines biological effect and therapeutic accessibility. An increase in IL-6, IL-1β, TNF-α, or selected chemokines cannot by itself identify the responsible population or establish whether the response is damaging, compensatory, or associated with repair. Single-cell and spatial datasets can nominate candidate SASP-producing populations, but shared inflammatory transcripts and transcript dropout preclude assigning senescence from these data alone; orthogonal protein, imaging, and functional validation is required (28, 29).

### 2.3 The p53–p21 and p16–RB axes define non-equivalent senescence programs

The p53–p21 and p16–RB axes are overlapping but non-equivalent routes into and through senescence. DNA damage commonly stabilizes p53 and induces p21, producing an early arrest that may permit repair, progress to senescence, or trigger apoptosis; p16 inhibits CDK4/6 and reinforces RB-dependent repression, often contributing to a more durable arrest. These pathways converge on cell-cycle control but differ by cell type, stress, and phase, and neither p53, p21, nor p16 alone identifies a uniform senescent population (30, 31). In human BM-MSCs, older donors showed higher p16 and p21 expression without a significant change in p53 transcript, illustrating why upstream and downstream readouts need not move together (18). Bone-specific perturbation strengthens the distinction. Selective clearance of p21-positive, but not p16-positive, cells prevented radiation-induced osteoporosis and marrow adiposity (15), and genetic removal of p21-positive osteochondroprogenitors and neutrophils accelerated fracture repair (16). In osteocytes, talin1 loss promoted nuclear p53 accumulation together with p16/p21-associated senescence, whereas osteocyte-specific p53 deletion rescued bone loss and impaired mechanoadaptation (24). Thus, p53 is an important stress integrator, p21 and p16 can mark different temporal or cellular programs, and a credible bone senotype requires multiple orthogonal markers plus functional evidence.

### 2.4 Senolytics eliminate selected persistent senescent-cell states

Senolytics induce apoptosis by disrupting survival pathways on which particular senescent cells depend. The original *in vitro* screen already demonstrated cell-selective susceptibility: dasatinib was more effective against senescent human preadipocytes, whereas quercetin was more effective against senescent endothelial cells, motivating their combination (32). Navitoclax targets BCL-2-family dependencies in several experimental senotypes (33), and fisetin shows senotherapeutic activity across cultured cells, human tissue models, and aged mice (34), although neither agent has established clinical skeletal efficacy. *In vivo* bone evidence is stronger: genetic or pharmacological clearance prevented age-related bone loss in mice (10), short-course dasatinib plus quercetin improved fracture healing in old mice (35), and bone-targeted delivery enhanced skeletal selectivity in senile osteoporosis models (36). Ex vivo activity should therefore be treated as a bridge, not a substitute, for bone-specific target engagement. Clinically, intermittent dasatinib plus quercetin did not reduce bone resorption in the overall cohort of a phase 2 trial in postmenopausal women (17), underscoring the need for biological stratification rather than indiscriminate senolysis.

### 2.5 Senomorphics suppress harmful senescent-cell outputs without cell killing

Senomorphics attenuate the SASP or related stress programs while leaving the cell alive, making them conceptually preferable when cell removal could disrupt tissue architecture or an evolving repair response. *In vitro*, metformin suppresses NF-κB-dependent inflammatory SASP genes (37), while rapamycin restrains the secretory phenotype through mTOR-regulated mechanisms (38). In aged mice, JAK inhibition reduces SASP-associated inflammation and improves physical function (39). These studies establish pharmacological tractability across *in vitro* and *in vivo* systems, but direct, cell-resolved evidence in aging bone remains limited. Senomorphics may be most appropriate when a maladaptive secretome dominates and the target cell remains structurally or temporally useful; they are less likely to suffice when a large persistent burden disrupts lineage function or niche occupancy. Senolytics and senomorphics are therefore distinct intervention classes, not interchangeable labels.

## 3 Senescent skeletal cells: cell-type-specific drivers of bone loss and repair failure

Skeletal cells translate age-related stress into lineage-specific defects and paracrine signals. Marrow stromal cells lose osteogenic competence and increasingly adopt adipogenic fates; osteoblast-lineage cells produce less matrix; osteocytes acquire DNA damage and abnormal remodeling signals; and osteoclast precursors encounter a niche that favors their differentiation. Recent evidence links these changes to senescence-associated stromal, osteocyte, adipocyte, and immune programs rather than to a single aging pathway. Together, the changes reduce bone formation, increase resorption and marrow adiposity, and compromise repair. A long-lived osteocyte embedded in mineralized matrix is unlikely to share the same vulnerabilities as a proliferative stromal progenitor or an injury-induced osteochondroprogenitor. Resolving these lineage-specific programs is essential for therapy (**Figure 3**).

**Figure 3:**
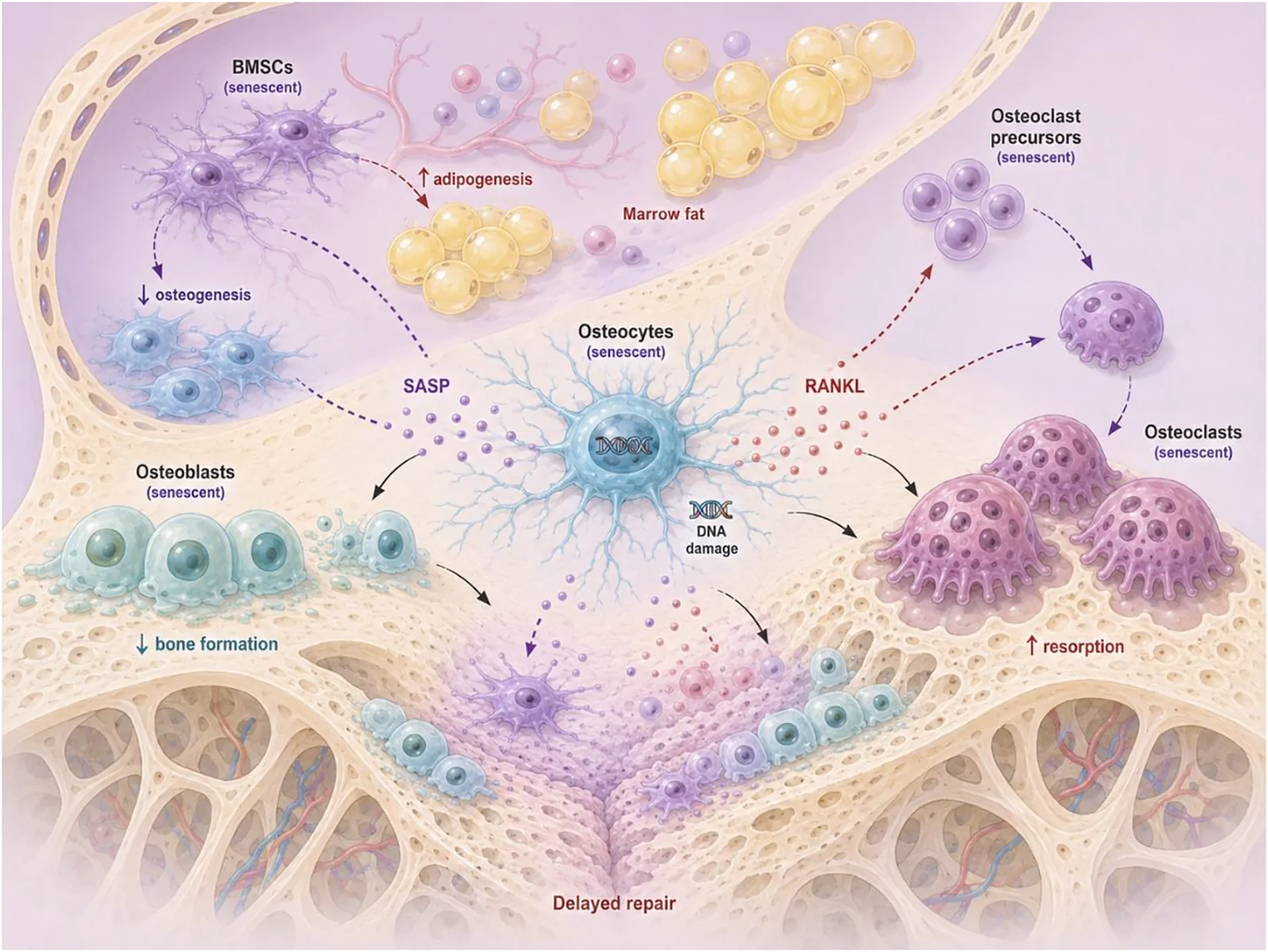
Senescent skeletal cells drive bone loss and repair failure through cell-type-specific mechanisms. This figure illustrates how senescence in skeletal-lineage cells reshapes the aging bone microenvironment. Senescent bone marrow stromal cells (BMSCs) show reduced osteogenic differentiation and increased adipogenic commitment, leading to impaired bone formation and marrow fat accumulation. Senescent osteoblast-lineage cells further weaken matrix production and anabolic bone remodeling. Senescent osteocytes act as central signaling hubs by accumulating DNA damage and releasing SASP factors and RANKL, thereby promoting osteoclastogenesis and local inflammatory remodeling. Osteoclast precursors respond to this senescent niche and differentiate into bone-resorbing osteoclasts, increasing skeletal resorption. Persistent senescence within the fracture callus and surrounding skeletal niche also interferes with progenitor function and delays bone repair. Together, these interconnected processes shift bone remodeling toward decreased formation, increased resorption, marrow adiposity, and impaired regeneration. All elements in this figure were originally drawn by the authors using Adobe Illustrator.

### 3.1 Senescent bone marrow stromal cells impair osteogenesis and favor marrow adiposity

Bone marrow stromal cells provide a major source of osteoblast-lineage cells in adult bone, making their functional aging a direct constraint on bone formation. Cells from older human donors show slower proliferation, reduced clonogenicity, increased oxidative stress, and higher p16/p21 and SA-β-gal readouts (18), while Osx1-expressing osteoprogenitors in mice accumulate DNA damage and senescence markers with age (41). These intrinsic changes contract the pool of competent osteogenic progenitors and reduce its responsiveness to anabolic or injury-related cues.

The consequences extend beyond progenitor exhaustion. Aging shifts skeletal stem and stromal cells away from osteogenesis and toward adipogenesis (7), thereby linking low bone formation with the expansion of marrow fat. Through the SASP, senescent stromal cells may also disrupt neighboring progenitors and immune populations (8), converting a cell-autonomous deficit into a niche-wide disturbance. Stromal senescence matters not only to the loss of osteoblast supply but also to the broader inflammatory and metabolic remodeling of marrow. Interventions aimed at this compartment may need to restore lineage competence while simultaneously limiting its maladaptive secretory output.

### 3.2 Bone–fat imbalance converts stromal aging into niche dysfunction

The osteoblast–adipocyte fate choice makes bone loss and marrow fat expansion two outputs of the same stromal imbalance. Aging and obesity increase marrow adipocyte accumulation and impair stem-cell-based hematopoietic and bone regeneration (42), while loss of the histone demethylase KDM4B accelerates stromal exhaustion, reduces bone formation, and increases marrow adiposity (43). More recent work identifies a senescent marrow-adipocyte program driven by PCLAF (23). Marrow adipocytes are heterogeneous and can support hematopoiesis in selected contexts; therefore, adipocyte abundance alone should not be equated with pathology. The actionable state is a loss of osteogenic competence combined with an inflammatory or senescence-associated adipocyte phenotype, ideally demonstrated by lineage, metabolic, and functional measures.

### 3.3 Osteoblast-lineage senescence weakens bone formation

Osteoblasts execute bone matrix synthesis and mineralization, and their decline is a defining feature of skeletal aging. The resulting reduction in bone formation reflects both fewer osteoblasts and impaired function among the cells that remain (7, 40). Cellular senescence offers one mechanistic link between upstream stress and this anabolic failure (5). Telomere dysfunction, for example, can induce arrest in osteoblast-lineage cells and impair differentiation and mineralization (44), whereas, in a cadmium-exposure model of osteoporosis, disruption of SIRT1–SOD2 signaling and mitochondrial homeostasis promoted osteoblast senescence (45). Osteoblast aging is more than a passive loss of activity; it represents an active stress response capable of altering the local environment. Secreted factors from senescent osteoblast-lineage cells may influence osteoclast formation, immune recruitment, and stromal-cell behavior (8), coupling reduced osteogenesis to increased inflammatory and resorptive signaling. Anabolic stimulation alone may consequently be insufficient unless the senescence-associated stress and secretory programs within this lineage are also controlled.

### 3.4 Senescent osteocytes connect bone aging with remodeling signals

Osteocytes are long-lived, matrix-embedded cells that coordinate mechanosensing, remodeling, and communication with bone-surface cells (46). Their longevity makes them particularly susceptible to cumulative damage, and senescent osteocytes are now recognized as a prominent component of aged bone (47). In old mice, osteocyte-enriched bone samples exhibit increased expression of senescence- and SASP-associated markers (47), whereas aging also induces *de novo* intracortical remodeling and cortical porosity (48). Mechanistically, osteocyte-derived RANKL is required for age-associated cortical bone loss, and senescence can induce RANKL expression within the osteocyte lineage (11), providing a direct route from cellular aging to osteoclast activation. Osteocyte influence also extends beyond the mineralized compartment. Experimental osteocyte ablation alters lineage commitment in bone and marrow and induces senescence-related changes (49). Senescent osteocytes may function as durable signaling hubs that couple cortical deterioration to broader immune and metabolic remodeling of the marrow niche.

### 3.5 Osteoclast precursors respond to the senescent skeletal niche

Osteoclasts derive from monocyte–macrophage precursors and execute the resorptive arm of remodeling. During aging, their differentiation is favored by increased RANKL, inflammatory cytokines, altered stromal support, and changes in immune-cell function (5, 7). Much of this effect may be non-cell-autonomous. SASP factors released by senescent skeletal and marrow populations alter bone locally and systemically (8), while senescent osteocytes enhance RANKL signaling and thereby support osteoclast formation (11). Osteoclast activation in aged bone should therefore be interpreted partly as a response to cells elsewhere in the niche rather than solely as intrinsic aging of the precursor. Because shared myeloid ancestry does not itself demonstrate a causal increase in resorption, age-associated myeloid bias should be regarded as a testable upstream contributor, not an established driver (21, 40).

### 3.6 Myeloid skewing may increase resorptive potential but is not sufficient evidence of causation

Aging shifts HSC composition and output toward myeloid-biased states, while osteoclasts arise from monocyte–macrophage precursors. This makes an expanded precursor supply a biologically plausible contributor to resorption, particularly in a niche rich in RANKL and inflammatory cytokines (21, 40). However, the chain from aged niche to HSC myeloid bias to osteoclast-mediated bone loss has not been established as a single causal sequence. A young niche only partly restores aged HSC function (21), and age-associated stromal inflammation can alter lymphoid and myeloid output (20), but neither result alone proves that myeloid skewing drives skeletal resorption. Lineage tracing and perturbation studies that separate precursor abundance from osteoclast differentiation and activity are needed.

### 3.7 Senescent skeletal cells delay fracture repair when they persist

Fracture repair requires an ordered progression from inflammation and progenitor expansion to cartilage formation, ossification, and remodeling. Senescent cells appear within the fracture callus, but their consequences depend on duration, lineage, and host age. In young mice, reducing the transient senescent-cell burden did not impair repair and instead accelerated healing (50), indicating that skeletal injury differs from settings in which senescence is indispensable for tissue closure. Aging shifts this balance further toward persistence. Senescent callus cells in old mice secrete TGF-β1, inhibit mesenchymal progenitors, and can be cleared by short-course dasatinib plus quercetin to improve healing (35). More recent work identifies p21-positive osteochondroprogenitors and neutrophils as a distinct injury-associated population; genetic removal of these cells suppresses senescence signatures and accelerates repair (16). The skeletal compartment accordingly contains multiple senescent states whose effects cannot be predicted from a single marker or time point. Their interaction with immune cells becomes the next critical layer of the osteoimmune response.

## 4 Senescent immune cells as active regulators of aging bone

Skeletal senescence develops within an immune-rich marrow environment. Aging immune populations do not merely accompany bone loss; they can alter inflammatory tone, osteoclastogenesis, progenitor behavior, and the capacity to resolve injury. T cells supply TNF-α, IL-17, and RANKL-related signals; macrophages shift between supportive and inflammatory states; neutrophils release state-specific mediators; and B and NK cells influence the RANKL/OPG axis or senescent-cell surveillance. Recent marrow and osteoimmune studies support these cell-specific changes while also emphasizing that their skeletal effects remain context- and model-dependent (22, 52, 53). These signals converge in the same niche as senescent skeletal cells, creating the potential for reciprocal reinforcement between immune dysfunction and tissue degeneration (**Figure 4**).

**Figure 4:**
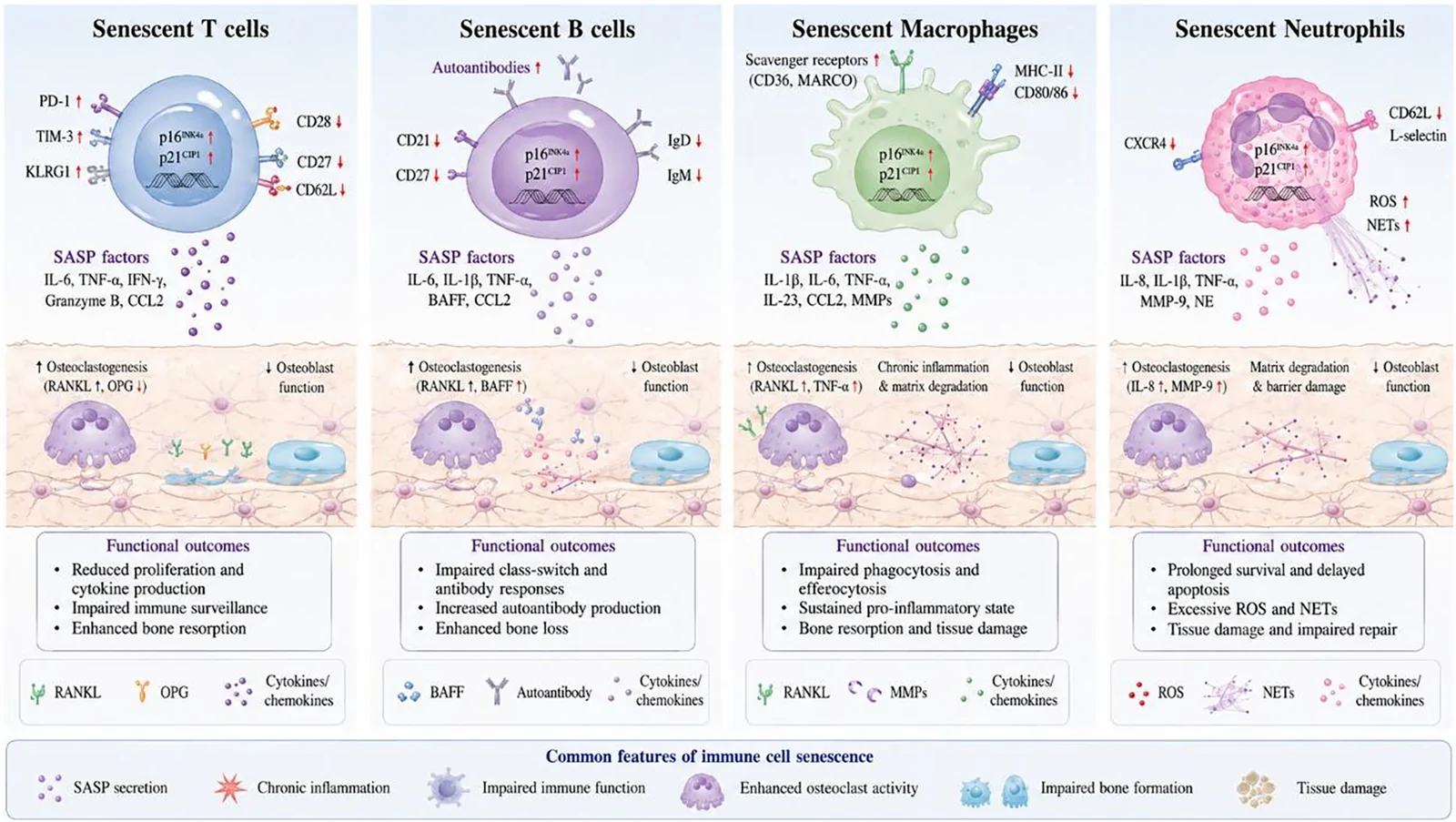
Senescent immune cells as active regulators of aging bone. The figure summarizes how senescence of major immune cell populations contributes to osteoimmune remodeling in aging bone. Senescent T cells show increased inhibitory or terminal differentiation markers, reduced co-stimulatory receptors, and enhanced production of inflammatory and osteoclastogenic mediators, thereby promoting bone resorption. Senescent B cells display altered antibody-related functions and disrupted RANKL/OPG-associated regulation, which may shift bone remodeling toward osteoclast activation. Senescent macrophages acquire a pro-inflammatory phenotype, impaired phagocytic capacity, and increased secretion of SASP factors, extracellular vesicles, and matrix-remodeling mediators, leading to chronic inflammation, progenitor dysfunction, and tissue damage. Neutrophils with senescence-associated or aging phenotypes may exhibit excessive ROS generation, NET formation, delayed clearance, and pro-inflammatory mediator release; canonical stable senescence *in vivo* remains unresolved. Together, these immune-derived SASP signals converge on the bone microenvironment, enhancing osteoclastogenesis, reducing bone formation, damaging the extracellular matrix, and sustaining a self-reinforcing osteoimmune senescence loop. All elements in this figure were originally drawn by the authors using Adobe Illustrator.

### 4.1 Senescent T cells link immune aging to osteoclastogenesis

T cells can either promote or restrain osteoclastogenesis, depending on activation state and cytokine output (54). Activated T cells express RANKL, and estrogen deficiency expands TNF-producing T cells in marrow, thereby increasing osteoclast formation and bone loss (55, 56). Aging and chronic stimulation further drive subsets toward senescence-like phenotypes (57). In rheumatoid arthritis, CD28-negative T cells accumulate and promote osteoclastogenesis more effectively than conventional CD28-positive cells (58), illustrating how immune aging can acquire direct skeletal consequences. Subset composition is equally important: Th17 cells act as osteoclastogenic helper cells and link adaptive immune activation to bone destruction (59), whereas IFN-γ can interfere with RANKL–RANK signaling and suppress osteoclast formation (54). T-cell senescence cannot be reduced to immune exhaustion; it changes the balance of signals that govern resorption and may contribute to bone loss beyond overt autoimmune disease.

### 4.2 Aged macrophages remodel the skeletal niche

Marrow macrophages regulate inflammation, efferocytosis, progenitor support, and osteoclast-lineage activity (51). Under physiological conditions they help maintain osteoblast function and coordinate repair, but aging erodes this supportive balance and favors persistent inflammatory states. Their influence is not confined to local cytokine production. Senescent marrow monocytes and macrophages can transmit aging signals to distant tissues through extracellular vesicles (52), positioning the marrow as a potential source of systemic senescence propagation. During skeletal repair, aged callus macrophages release grancalcin, induce senescence in skeletal stem and progenitor cells, and impede healing (13). Macrophage aging connects defective immune resolution with both local regenerative failure and broader organismal aging. It may represent a particularly important therapeutic node when inflammation and progenitor dysfunction coexist.

### 4.3 Neutrophils and innate inflammatory aging contribute to skeletal decline

Neutrophils merit attention because distinct age- and injury-associated states have been linked experimentally to bone loss and failed repair; grancalcin is one tractable mediator, not an exhaustive account of their biology. Li et al. showed that aged marrow neutrophils and macrophages released grancalcin and that recombinant protein or myeloid-cell manipulation altered skeletal aging (12). A separate study identified an age-expanded TGFβ1+CCR5+ neutrophil subset in male mouse marrow; transfer and pathway experiments linked the subset to bone loss, while CCR5 inhibition with maraviroc reduced its abundance and increased bone mass (22). After fracture, p21-positive neutrophils appear alongside osteochondroprogenitors, and genetic clearance of the combined p21-positive population accelerates repair (16). Together, these data support multiple neutrophil-associated mediators and states rather than a grancalcin-only model.

Attribution and terminology nevertheless require restraint. Zou et al. specifically implicated aged macrophage-derived grancalcin in skeletal stem/progenitor-cell senescence during fracture healing; that result should not be recast as evidence for a neutrophil-dominant source (13). Moreover, the short lifespan and terminal differentiation of neutrophils make bona fide cellular senescence difficult to establish. p21 expression, SASP-like mediators, or altered function alone do not demonstrate a stable senescent arrest. We therefore use ‘senescence-associated neutrophil state’ or ‘neutrophil aging’ unless multiparametric and functional evidence supports senescence; whether neutrophils undergo canonical cellular senescence *in vivo* remains unresolved.

### 4.4 B cells and NK cells add another layer to osteoimmune senescence

B cells contribute to bone homeostasis through the RANKL/OPG system (60). In aged mice, production of both mediators increases, implying that aging modifies the balance between pro- and anti-resorptive B-cell functions. Because OPG acts as a decoy receptor for RANKL, changes in B-cell-derived OPG may materially affect age-related osteoclastogenesis (61). Clinical observations in chronic HIV infection, where dysregulated B-cell RANKL and OPG expression correlates with reduced bone mineral density, further support this immune–skeletal connection (62). Direct evidence that senescent B cells drive primary skeletal aging remains limited, however, and should not be inferred from altered cytokine production alone (60). NK cells add a surveillance dimension. They can promote osteoclastogenesis in inflammatory arthritis (63), but they also recognize NKG2D ligands on senescent cells and contribute to their clearance (64). Determining whether NK-cell aging permits senescent cells to persist in bone is an important unresolved question.

### 4.5 Immune-derived SASP factors integrate bone loss, marrow aging, and repair failure

Cytokines, chemokines, proteases, and extracellular vesicles released by senescent immune cells can alter skeletal progenitors, osteoblasts, and osteoclast precursors (12, 52). The immune-derived SASP is a functional element of the marrow niche rather than a distant systemic influence (51). Several pathways illustrate this integration: macrophage extracellular vesicles propagate senescence, grancalcin from aged neutrophils and macrophages suppresses skeletal homeostasis, and senescent T-cell populations enhance osteoclastogenesis through inflammatory and RANKL-related signals (58). Their convergence creates a self-reinforcing osteoimmune circuit (14) that is unlikely to be reversed by targeting skeletal cells alone. RANKL blockade can restrain resorption (61), but broader correction may require immune modulation, restoration of surveillance, and support for bone formation. Understanding how these cellular interactions are organized within the aging marrow niche is essential.

## 5 The senescent bone marrow niche: crosstalk among bone, immunity, hematopoiesis, and metabolism

The bone marrow is an integrated tissue ecosystem rather than a passive cavity in which bone and blood cells coexist. Stromal, hematopoietic, immune, adipose, endothelial, and neural components jointly regulate skeletal remodeling and blood-cell production. Aging disrupts each compartment: stromal cells lose osteogenic support, HSCs show reduced regenerative capacity and myeloid-biased output, marrow adipocytes expand, and vascular and neural inputs deteriorate. Recent cellular and niche studies document these parallel changes and their partial interdependence. Because many links remain associative, the aged marrow is best viewed as a coupled osteoimmune–hematopoietic–metabolic system in which stromal dysfunction, inflammatory signaling, hematopoietic drift, vascular and neural decline, and marrow fat can reinforce skeletal deterioration (**Figure 5**).

**Figure 5:**
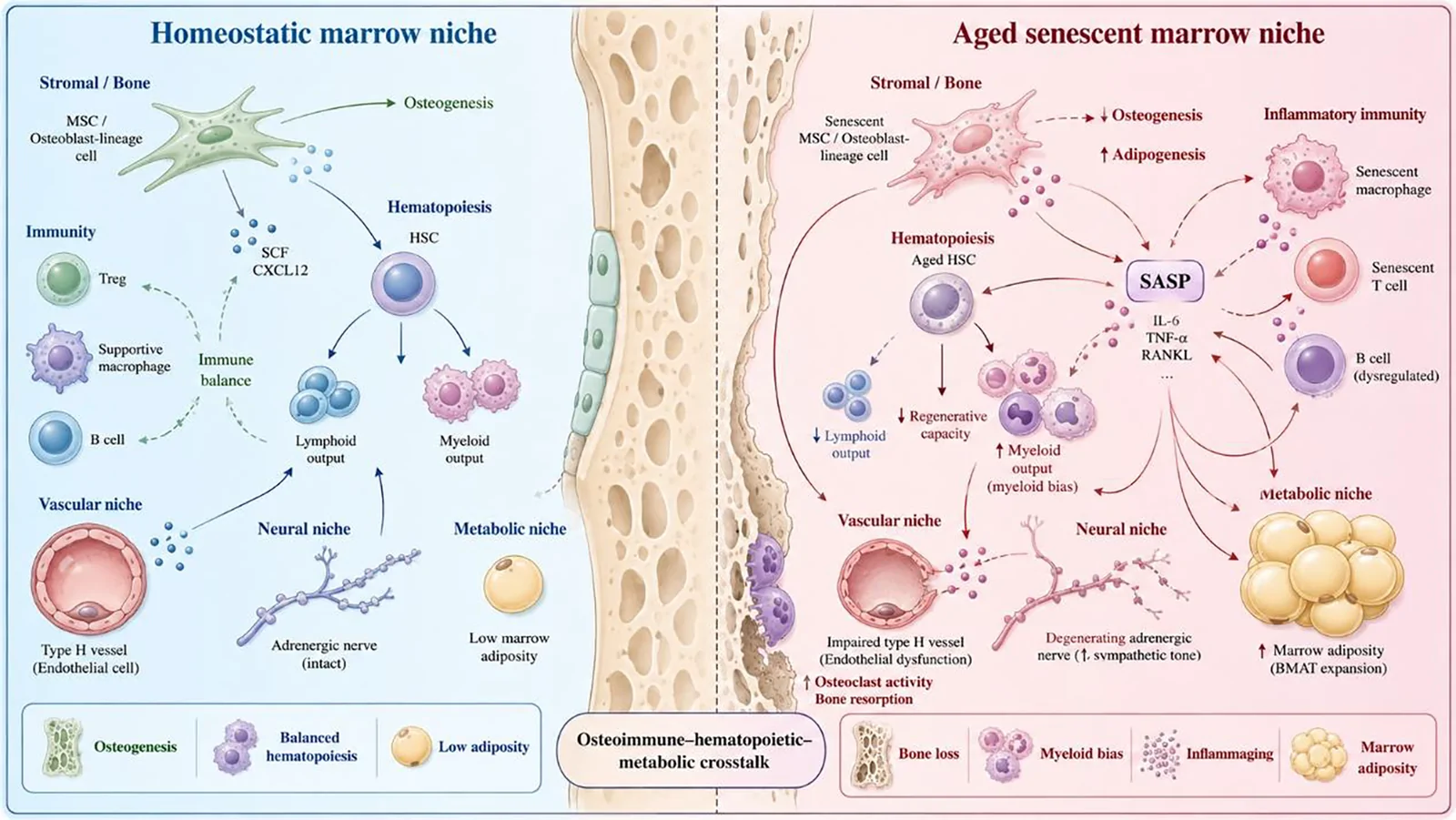
Senescent bone marrow niche as an osteoimmune–hematopoietic–metabolic ecosystem. The figure compares the homeostatic marrow niche with the aged senescent marrow niche. Under physiological conditions, mesenchymal stromal cells and osteoblast-lineage cells support osteogenesis and maintain hematopoietic stem cell function through niche factors such as SCF and CXCL12. Balanced immune regulation, intact type H vessels, adrenergic nerve fibers, and low marrow adiposity together sustain coordinated bone remodeling and balanced lymphoid–myeloid hematopoiesis. During aging, stromal and osteoblast-lineage cells acquire senescent features, lose osteogenic capacity, and shift toward adipogenic programs. At the same time, aged hematopoietic stem cells show reduced regenerative capacity and myeloid-biased differentiation. Senescent immune cells, including macrophages and T cells, amplify inflammatory signaling through SASP factors such as IL-6, TNF-α, and RANKL, thereby promoting osteoclast activity and bone resorption. Expansion of bone marrow adipose tissue, endothelial dysfunction, and degeneration of adrenergic neural inputs further weaken niche support. These alterations reinforce each other and form a feed-forward osteoimmune–metabolic loop that drives bone loss, inflammaging, marrow adiposity, immune dysregulation, and impaired skeletal regeneration. All elements in this figure were originally drawn by the authors using Adobe Illustrator.

### 5.1 The bone marrow niche links skeletal remodeling with immune aging

HSC maintenance depends on signals from perivascular mesenchymal cells, endothelial cells, osteolineage populations, and other local constituents, with SCF and CXCL12 providing core niche support (67). Many of these same cells also regulate bone remodeling and immune activity (65), placing hematopoiesis within, rather than adjacent to, skeletal biology. Aging weakens HSC regenerative capacity and alters lineage output (66), while inflammatory remodeling of the local environment further compromises niche support (68). This arrangement creates a reciprocal circuit: senescent skeletal and stromal populations influence immune-cell production, and the resulting aged immune cells return inflammatory and pro-resorptive signals to bone. Bone and immune aging should accordingly be studied as coupled events within a shared anatomical niche.

### 5.2 Stromal niche aging drives osteogenic failure and hematopoietic drift

Marrow stromal cells organize both osteogenesis and hematopoiesis. They generate osteoblast-lineage cells and provide essential HSC support (67), yet aging redirects stromal fate toward adipogenesis and away from bone formation (23, 43). In parallel, aged HSCs show myeloid-biased output and reduced regenerative capacity (21). Transplantation into a young environment only partly restores aged HSC function, supporting contributions from both cell-intrinsic damage and extrinsic niche signals. These observations do not by themselves establish that an aged niche causes myeloid bias or that myeloid bias causes bone resorption; each link requires direct perturbation and skeletal readouts. Niche-directed therapy may therefore need to restore stromal support while addressing persistent intrinsic HSC aging.

### 5.3 Marrow adiposity converts metabolic aging into skeletal and immune dysfunction

Marrow adiposity rises with age and accompanies the shift of mesenchymal stromal cells from osteoblast toward adipocyte differentiation (42, 43). Marrow adipocytes are not passive space-filling cells: their effects on hematopoiesis vary by skeletal site and physiological context, and bone marrow adipose tissue functions as an endocrine and immune-metabolic compartment (9, 69). Cellular senescence adds another layer. PCLAF promotes marrow-adipocyte senescence and contributes experimentally to skeletal aging (23), indicating that adipogenic drift can generate active degenerative signals. Marrow fat is therefore both a marker of lost osteogenic potential and, in selected states, a source of metabolic and senescence-associated stress; abundance alone is not sufficient to infer dysfunction.

### 5.4 Vascular and neural niche aging weakens bone-marrow communication

Vascular integrity is required for both hematopoiesis and bone formation. Endothelial cells support HSC function and regeneration (70), while specialized type H vessels couple angiogenesis to osteogenesis (71). In humans, the abundance of type H vessels is associated with bone quality and may provide an early indicator of skeletal loss (72). Their age-related decline, together with loss of adjacent osteoprogenitors, weakens the vascular–osteogenic unit and contributes to defective marrow support (68, 71). Neural regulation is similarly relevant. Degeneration of adrenergic nerves remodels marrow stromal cells and accelerates aging of the HSC niche (73). Vascular and neural aging disrupt the communication channels that coordinate HSCs, immune cells, stromal populations, and bone-forming cells, broadening osteoimmune senescence beyond the conventional osteoblast–osteoclast axis.

### 5.5 Senescent niche cells create feed-forward osteoimmune-metabolic loops

These cellular defects become most consequential when they form feed-forward loops. Senescent bone cells impair osteogenesis and increase osteoclastogenic signaling (10); aged immune cells release grancalcin or extracellular vesicles that propagate skeletal or systemic senescence (12, 52); marrow adipocytes can add metabolic and inflammatory stress; and vascular or neural decline removes trophic support. The result may be niche-level failure rather than an isolated defect in one lineage. Anti-resorptives can suppress osteoclasts while leaving inflammatory and stromal abnormalities intact, whereas an anabolic stimulus may be blunted by a hostile marrow environment. These therapeutic inferences remain hypotheses until the dominant compartment is identified and perturbed; current clinical and review evidence supports stratification, not a universal causal hierarchy (14, 17).

### 5.6 Single-cell and spatial tools can nominate—but not by themselves define—the senescent marrow niche

Single-cell and spatial approaches are valuable for mapping marrow populations and neighborhoods (74), but they do not solve senescent-cell identification by themselves. Senescent cells are often rare, enlarged, fragile, and adhesive, so dissociation and quality-control filters can preferentially remove them. CDKN2A/p16 and CDKN1A/p21 transcripts are sparse and subject to dropout, while many SASP genes are shared with activated, injured, or differentiating cells. Postmitotic osteocytes further complicate inference from cell-cycle arrest. Gene sets such as SenMayo improve sensitivity across datasets but are not universal, tissue-independent identifiers (28). Spatial transcriptomics adds location but may have limited molecular capture or multicellular spots. Accordingly, single-cell or spatial data should be used to nominate candidates, followed by at least two orthogonal senescence domains—such as arrest-pathway activation, persistent DNA damage, lysosomal or morphological change, and a compatible secretome—together with exclusion of quiescence and acute activation, imaging or protein confirmation, and, where feasible, lineage tracing or functional perturbation (29). This staged validation makes spatial senotyping informative without overpromising specificity.

## 6 Disease-specific osteoimmune senescence: shared mechanisms and divergent programs

The architecture of osteoimmune senescence differs across skeletal diseases. Osteoporosis is dominated by systemic bone loss and marrow-niche dysfunction; osteoarthritis involves cartilage, synovium, subchondral bone, and immune cells within the joint; rheumatoid arthritis superimposes premature immune and stromal aging on autoimmunity; periodontitis combines microbial challenge with aging of periodontal and alveolar bone tissues; and delayed fracture healing reflects failure to resolve an injury-associated program. Recent disease-specific studies support shared SASP amplification, osteoclast activation, impaired progenitor function, and defective resolution, while also showing that the dominant cells, compartments, and timing differ. These diseases are therefore most informative when compared through both common mechanisms and divergent senescence landscapes (**Figure 6**).

**Figure 6:**
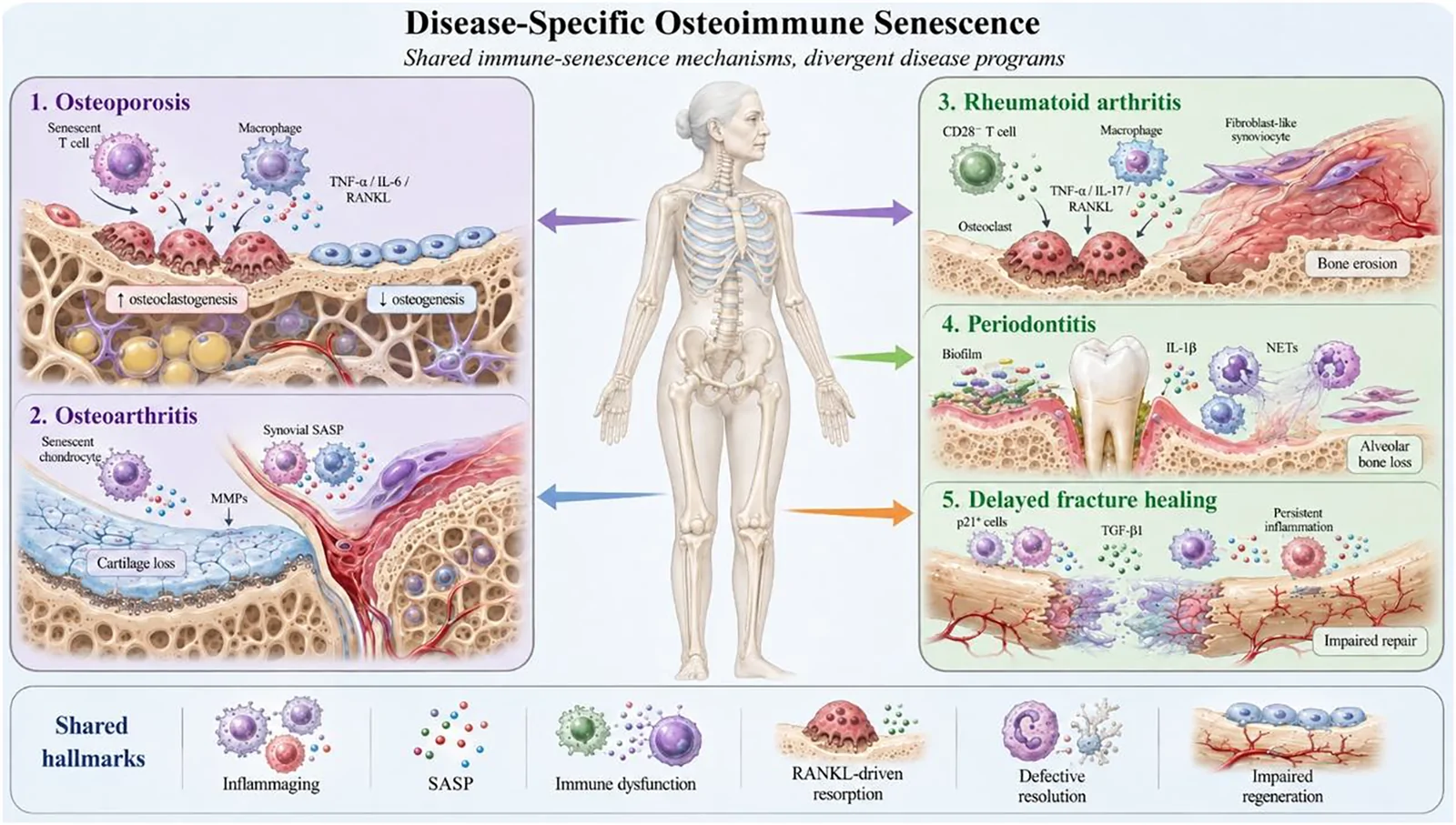
Disease-specific osteoimmune senescence in aging-related bone diseases. This schematic illustrates shared and disease-specific mechanisms by which osteoimmune senescence contributes to skeletal pathology. In osteoporosis, senescent osteocytes, stromal cells, T cells, and macrophages promote RANKL-driven osteoclastogenesis, suppress osteogenesis, and increase marrow adiposity, leading to systemic bone loss. In osteoarthritis, senescent chondrocytes, synovial fibroblasts, and subchondral bone cells produce SASP factors, matrix-degrading enzymes, and inflammatory mediators that drive cartilage degeneration, synovitis, and abnormal bone remodeling. In rheumatoid arthritis, premature senescence of immune cells and fibroblast-like synoviocytes amplifies TNF-α, IL-17, RANKL, chemokines, and MMPs, thereby enhancing synovial inflammation and focal bone erosion. In periodontitis, microbial challenge interacts with senescent periodontal cells, macrophages, neutrophils, and osteoclast precursors to sustain inflammation and alveolar bone loss. In delayed fracture healing, persistent senescence in progenitor, endothelial, immune, and chondrogenic compartments impairs inflammatory resolution, angiogenesis, callus remodeling, and tissue regeneration. Despite divergent tissue contexts, these diseases share core hallmarks, including inflammaging, SASP activation, immune dysfunction, RANKL-mediated resorption, defective resolution, and impaired repair. All elements in this figure were originally drawn by the authors using Adobe Illustrator.

### 6.1 Osteoporosis represents systemic osteoimmune senescence

Osteoporosis most clearly illustrates systemic osteoimmune senescence. Senescent cells accumulate in aging bone, and their genetic or pharmacological clearance prevents bone loss in old mice, supporting a causal rather than merely associative role (10). The osteoporotic skeleton also bears a strong immune imprint. Estrogen deficiency increases inflammatory signaling and osteoclast formation (55), in part through expansion of TNF-producing T cells in marrow (56), while senescent T-cell subsets can further enhance osteoclastogenesis (58). Osteoporosis reflects more than an imbalance between mature osteoblasts and osteoclasts; it arises within a marrow environment marked by stromal dysfunction, immune activation, and reduced osteogenic capacity (5). Yet the negative overall result of a phase 2 dasatinib-plus-quercetin trial in postmenopausal women shows that senescence is unlikely to be a uniform therapeutic target (17). Clinical use will require biomarkers that identify the patients and skeletal compartments in which senescent cells exert a dominant role.

### 6.2 Osteoarthritis shows tissue-compartment-specific senescence

Osteoarthritis (OA) affects the entire joint rather than cartilage alone, encompassing synovium, subchondral bone, meniscus, fat pad, and immune populations (77). Senescent cells and aging-associated inflammatory programs across these compartments contribute to matrix degradation, persistent inflammation, and pain (77, 78). Their effects are spatially distinct: senescent chondrocytes produce matrix-degrading enzymes and inflammatory mediators, while senescence in synovial and stromal compartments can sustain local inflammation and immune recruitment; abnormal subchondral remodeling feeds mechanical and inflammatory stress back to cartilage (77). Local clearance of senescent cells attenuates post-traumatic OA and promotes a regenerative joint environment in experimental models (79), providing proof of principle for anatomically restricted therapy. Unlike systemic osteoporosis, the relevant window in OA is likely to depend on the joint compartment, injury history, mechanical loading, and local inflammatory state.

### 6.3 Rheumatoid arthritis combines autoimmunity with premature senescence

Rheumatoid arthritis (RA) is a chronic autoimmune disease in which activated immune cells drive synovitis, osteoclastogenesis, and focal erosion (80). RANKL expression by activated T cells provides a direct mechanistic link between adaptive immunity and osteoclast formation. Senescence adds a further layer: CD4^+^CD28^-^ T cells are enriched in RA and display strong osteoclastogenic activity (58), indicating that premature immune aging can directly intensify skeletal damage.

These senescence-like T cells promote osteoclast differentiation more effectively than conventional CD28-positive counterparts and may contribute to both focal erosion and systemic bone loss (58). Premature stromal senescence is also evident in RA synovium (81), where TNF-α and oxidative stress can accelerate fibroblast arrest and increase pro-inflammatory mediator production. Autoimmunity, cytokine exposure, and stromal remodeling thus reinforce one another within a persistent synovial circuit. This disease context differs fundamentally from primary osteoporosis: senescence-directed treatment in RA would probably need to complement, rather than replace, immune suppression or cytokine blockade.

### 6.4 Periodontitis links microbial inflammation with alveolar bone senescence

Periodontitis offers a distinct setting in which microbial challenge initiates disease within an aging host. Immune dysregulation increases susceptibility with age (82), and continuous exposure to bacterial products sustains inflammation in the periodontal niche (75). This environment can induce or amplify senescence in resident tissues. Senescent osteocytes worsen age-associated periodontal disease by increasing inflammation and restricting regeneration, thereby converting transient microbial injury into persistent alveolar bone loss (83). Human lesions likewise show increased p16, lipofuscin, and β-galactosidase staining, with senescence-like features enriched in fibroblasts and macrophages relative to healthy sites (76). Periodontitis is best understood as an interaction between dysbiosis-driven inflammation and an aged, poorly resolving tissue response. Effective treatment may need to reduce microbial burden while also addressing the senescence-associated inflammatory memory that remains in the host niche.

### 6.5 Delayed fracture healing reflects failed resolution of senescence

Fracture healing differs from chronic skeletal degeneration because senescence emerges within a time-limited regenerative program. Senescent cells transiently accumulate in murine callus, yet lowering their abundance does not impair repair and can accelerate it (50). In old animals, persistence becomes more clearly pathogenic: callus cells secrete TGF-β1, suppress mesenchymal progenitors, and respond to short-course dasatinib plus quercetin with improved healing (35). The identification of p21-positive osteochondroprogenitors and neutrophils after fracture further refines this model; genetic clearance of these populations reduces senescence-associated signatures and accelerates repair (16). Delayed healing thus reflects, at least in part, failure to terminate specific injury-associated cell states. It also demonstrates why the effect of senescence cannot be classified as uniformly beneficial or harmful without considering time, lineage, and clearance efficiency.

### 6.6 Shared pathways do not mean identical senescence states

Across these diseases, SASP factors amplify inflammation, impair stromal function, and support osteoclastogenesis (5). Senescent T cells provide RANKL-related and inflammatory signals (58), TNF-α from T cells promotes bone resorption (55), and Th17 cells couple immune activation to osteoclast formation (59). Senescent skeletal cells suppress osteogenesis while remodeling their surroundings (10). This shared molecular vocabulary does not imply a shared cellular state. Systemic aging and estrogen loss dominate osteoporosis (5), whereas mechanical injury and multicompartment joint pathology shape OA (77). Autoimmunity accelerates immune and stromal senescence in RA (58), microbial exposure interacts with host aging in periodontitis (75), and unresolved callus senescence limits fracture repair (35). A single senolytic regimen is unlikely to fit all conditions. Disease stratification should incorporate senescent-cell burden, dominant lineage, immune phenotype, anatomical compartment, and stage—variables that also define the therapeutic windows considered below.

## 7 Therapeutic windows: from broad senolysis to precision targeting of osteoimmune senescence

A precision therapeutic window is operational only if four questions are answered before treatment: Is senescence a dominant and actionable process? Which population and compartment are implicated? Is the state transient or persistent? Which intervention can engage that state with an acceptable therapeutic index? **Table 2** converts these questions into a provisional research framework. The proposed rules are deliberately conservative because no universal senescence biomarker or clinically validated cutoff exists (29).

**Table 2:** Provisional biomarker-guided stratification framework for senescence-targeted intervention in aging bone.

Decision node	Candidate measurements	Proposed research rule	Matched action	Key limitation
1. Is senescence dominant?	At least two domains: p16/p21–RB arrest; γH2AX/53BP1 or telomere-associated foci; SA-β-gal/GLB1 or lipofuscin; composite SASP; site dysfunction	Proceed only when ≥2 orthogonal domains exceed age-, sex-, site-, and assay-matched reference limits and co-localize with dysfunction or persist on repeat sampling	If negative, prioritize established anti-resorptive, anabolic, anti-inflammatory, antimicrobial, or mechanical care; if positive, phenotype further	No universal clinical cutoff; SASP and single markers are non-specific (29)
2. Which cell and site dominate?	Flow/IHC or multiplex imaging plus lineage markers; marrow aspirate/biopsy where justified; marrow-fat MRI; joint or callus imaging; single-cell/spatial nomination (29, 74)	Require concordance across ≥2 modalities and anatomical co-localization; do not assign a senotype from transcriptomics alone	Systemic marrow disease: systemic or bone-targeted delivery; single joint/callus: local delivery; immune-dominant state: surveillance or immune-directed strategy (36, 79, 85)	Sampling error and poor access to deep skeletal compartments
3. Transient or persistent?	Time from fracture/injury, serial burden/SASP measures, healing stage, symptoms, turnover markers	Declining early repair-associated signal: observe or consider senomorphic control; plateau/rise beyond the expected disease-specific resolution period: evaluate clearance (16, 35, 50)	Avoid early indiscriminate senolysis; consider short, stage-defined senolytic exposure only for persistent pathogenic states	Normal resolution intervals and thresholds require prospective definition
4. Is the target drug-susceptible and safe?	Ex vivo kill or SASP-suppression assay where feasible; survival-pathway profile; CBC/platelets, hepatic/renal function, interactions (32, 33)	Illustrative preclinical go-rule: ≥50% reduction of multi-marker-positive target cells with ≥80% viability of matched non-senescent cells; prespecify toxicity limits	Senolytic for dispensable, persistent, susceptible cells; senomorphic for essential cells with harmful SASP; stop if selectivity is inadequate (37–39)	The 50%/80% values are proposed experimental thresholds, not validated clinical cutoffs
5. Is systemic senolysis justified?	T-cell p16_variant 5, plasma SASP score, burden across sites, clinical phenotype	Use the highest prespecified assay stratum only as trial enrichment; the highest-tertile signal in the D+Q study must not be treated as a diagnostic threshold (17, 84)	Clinical-trial enrollment; intermittent systemic dosing with target-engagement monitoring	Exploratory subgroup evidence; requires prospective validation
6. Has target engagement occurred?	Repeat ≥2 senescence domains, toxicity, bone-turnover markers, site-specific imaging and function (17, 29, 84)	Continue only if biomarker change exceeds assay variation/least significant change in ≥2 domains, is directionally concordant with skeletal outcomes, and toxicity remains acceptable	Continue, switch class/delivery, or stop according to a prespecified adaptive protocol	Biomarker decline is not itself proof of fracture or functional benefit

CBC, complete blood count; D+Q, dasatinib plus quercetin; IHC, immunohistochemistry; MRI, magnetic resonance imaging; SASP, senescence-associated secretory phenotype. All numerical rules in this table are proposed for prospective research protocols and require assay-, disease-, and site-specific validation before clinical use.

### 7.1 Establish whether senescence is dominant and actionable

A single positive p16, p21, SA-β-gal, or circulating cytokine result is insufficient. The minimum evidentiary unit should combine two orthogonal senescence domains with a spatially or temporally linked skeletal abnormality (29). In accessible research settings, T-cell p16_variant 5 and a correlated plasma SASP panel are candidate enrichment tools; both identified the same exploratory high-burden subgroup in the postmenopausal D+Q trial, but neither has a validated diagnostic cutoff (17, 84). The highest tertile used in that study is cohort-defined, not a threshold transferable to another population. If the composite is negative or equivocal, established disease-specific treatment should take precedence over senotherapy.

### 7.2 Resolve the dominant population, compartment, and stage

After a burden signal is established, lineage and location determine the plausible window. A persistent p21-associated population after radiation differs from a p16-associated osteocyte state (15), and an early fracture callus differs from chronic osteoporotic marrow (16, 35). Transcriptomic or spatial data can nominate a population, but flow cytometry, multiplex imaging, protein or damage markers, and functional perturbation should confirm it. Longitudinal sampling is essential when the biology is expected to resolve: a falling early injury-associated signal favors observation or reversible SASP control, whereas persistence beyond a prespecified disease-specific interval supports evaluation of clearance. These are stage-specific hypotheses that require prospective calibration, not universal time cutoffs.

### 7.3 Match intervention class and delivery to the dominant mechanism

Persistent, dispensable cells with demonstrated drug susceptibility are candidates for intermittent senolysis; essential or repair-associated cells with a harmful secretome favor senomorphic suppression. Immune-surveillance failure may justify immune-directed strategies: NKG2D-dependent recognition supports the concept (64), and NKG2D-CAR T cells clear senescent cells in preclinical models, although skeletal translation remains remote (85). Delivery should follow anatomy. Bone-targeted senolytics improve skeletal selectivity in senile osteoporosis models (36), while intra-articular clearance provides proof of principle for localized osteoarthritis (79). Standard anti-resorptive, anabolic, anti-inflammatory, antimicrobial, and rehabilitative therapies remain the clinical foundation; senotherapy should be tested as a biomarker-selected adjunct rather than a general anti-aging treatment.

### 7.4 Confirm target engagement and reassess the window

An intervention should continue only when the intended multi-marker population or SASP program changes beyond assay variation, toxicity remains acceptable, and skeletal measures move in a concordant direction. Short-term pharmacodynamic readouts may include a validated composite burden score and bone-turnover markers; imaging, pain, function, falls, and fractures require longer follow-up. The negative overall primary result of the phase 2 D+Q trial shows why *post hoc* subgroup signals cannot establish efficacy (17). Prospective enrichment, prespecified stopping rules, and replication are necessary before a candidate window can guide care.

### 7.5 Traditional Chinese medicine as a hypothesis-generating adjunct

Traditional Chinese medicine should be held to the same cell-resolved and clinical standards. A 240-participant trial reported increased bone mineral density after one year of QiangGuYin, but surrogate outcomes, formulation standardization, and replication limit inference (86); an appraisal of 20 guidelines found heterogeneous methods and recommendations (87). Ginsenoside Rg1 and icariin show preclinical anti-senescence, regenerative, or senomorphic activity in stromal or osteoblast models (88–90), but these findings do not demonstrate senolysis or validate syndrome patterns as biological senotypes. Such approaches should not replace established osteoporosis care. Their most rigorous role is as standardized, exposure-defined candidates in preregistered trials that test whether a formulation changes a prespecified senescence composite and a clinically meaningful skeletal outcome.

## 8 Conclusions and priorities

Osteoimmune senescence is most useful when it distinguishes rather than aggregates: p16- and p21-associated populations are not interchangeable; immune, stromal, adipose, and osteolineage states differ by site and stress; and an early repair response is not equivalent to persistent degeneration. The immediate priority is to validate multiparametric, site-aware biomarkers and prospective decision rules that connect a dominant cell state to intervention class, delivery route, and stage. Until then, senolytics, senomorphics, and immune-clearance strategies should remain biomarker-guided research tools used alongside established skeletal therapy. This operational, falsifiable framework—not a broader catalog of inflammaging pathways—is the principal contribution of the review.
